# 

**Published:** 1846-02

**Authors:** 


### CONTENTS

OF NO. II.

---

### ORIGINAL COMMUNICATIONS.

### ESSAYS AND CASES.

ART. I.—A Case of Uterine Hydatids; their expulsion attended by alarming Hæmorrhage; Recovery. By ISAAC EDWARDS, M.D., of Leavenworth, Indiana. - - 93

ART. II.—A Case of Abortion, the fœtus having been retained four months after its death. By DR. THOMAS C. OSBORNE, of Erie, Ala. - - - - - - - 98

### REVIEWS.

ART. III.—Urinary Deposits, their Diagnosis, Pathology and Therapeutical Indications. By GOLDING BIRD, A. M., M.D., Assistant Physician to, and Lecturer on Materia Medica at Guy's Hospital; Licentiate of the Royal College of Physicians; late President of the Westminster Medical Society; Fellow of the Linnean Society of London; Corresponding Member of the Philosophical Institute of Basle, of the Philosophical Society of St. Andrews, of the Medical Society of Hamburgh, &c., &c. - - - - - - 100

ART. IV.—Elements of Pathological Anatomy; Illustrated by colored Engravings and 250 Woodcuts. By SAMUEL D. GROSS, M.D., Professor of Surgery in the Medical Institute of Louisville; Late Professor of Pathological Anatomy in the Medical Department of the Cincinnati College; Surgeon to the Louisville Marine Hospital, &c., &c. Second Edition, thoroughly revised and greatly enlarged. - - - 112

ART. V.—On Diseases of the Liver. By GEORGE BUDD, M.D., F.R.S., Professor of Medicine in King's College, London; and Fellow of Caius College, Cambridge. With Colored Plates and numerous Woodcuts. - - - - - - - - - - - 134

SELECTIONS FROM AMERICAN AND FOREIGN JOURNALS.

Case of Intermittent Fever complicated with Purpura Hæmorrhagica: cured by Quinine and Nitric Acid. - - - 141
Hydrocele Cured by Electricity. - - - - - - 143
Insanity in a Boy four years of age, following Fright. - 143
Hay Asthma. - - - - - - - - - - 143
Chorea. - - - - - - - - - - - - 144
Fatal Vaccinia during Whooping Cough. - - - - - 144
Epilepsy. - - - - - - - - - - - 145
Hemiplegia. - - - - - - - - - - - 145
Quinine—its effect upon the Spleen, Liver, &c. - - 145
Uterine Hæmorrhage. - - - - - - - - - 146
The action of Sulphuric Ether on an Enlarged Spleen. - 147
Convulsions. - - - - - - - - - - - 147
Cases of Labor in which the presentations are obscure. - 148
Needle found in the Heart of a Cow. - - - - - 149
Iodide of Iron. - - - - - - - - - - 149
Puberty in the African. - - - - - - - - 150
Dropsical Affections successfully treated with Sugar. - - 151
Case of Lodgement and Retention of a Foreign Body in a Bronchus for the space of 60 years: its expulsion. - 151
Case of Suspension of the Mental Faculties. - - - - 153
Poisoning by Urine. - - - - - - - - - 158
French Medical Congress. - - - - - - - - 160
Serres' Address on opening the Medical Congress of Paris. . 161
Effects of Lightning. - - - - - - - - - 162
Turpentine in Large Doses in Purpura Hæmorrhagica. - 163
Hereditary Predisposition. - - - - - - - - 169
Confident Expectation in the Cure of Disease. - - - 170
Electricity in Exciting Uterine Action during Delivery. - 171
Rapid Death from Oxalic Acid. - - - - - - 172
The Late Dr. James Johnson. - - - - - - - 172

ORIGINAL INTELLIGENCE.

Georgia Lunatic Asylum. - - - - - - - - 177
Introductory Lectures. - - - - - - - - - 179
A Suit for Fees by a Clairvoyant. - - - - - - 180
Mysteries of Tobacco. - - - - - - - - . 181
Vestiges of Creation. - - - - - - - - - 182
Epidemic Small-Pox. - - - - - - - - - 183
Books Received. - - - - - - - - - - 183
Dr. Borland's Essay on Milksickness. - - - - - 184

